# Supplementary material for: Polysialic Acid Sustains the Hypoxia-Induced Migration and Undifferentiated State of Human Glioblastoma Cells
Source: Int J Mol Sci. 2022 Aug 24;23(17):9563. doi: 10.3390/ijms23179563 (PMC9455737; doi:10.3390/ijms23179563)
Supplement: Supplementary file 1 [file ijms-23-09563-s001.zip › Figures S1-5.pdf]

Supplementary figures

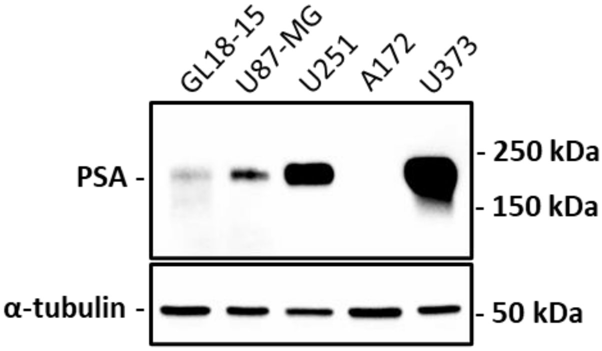

Figure S1 – PSA expression in human GBM cultures. Western blot analysis showing the expression of PSA in four human GBM cell lines (U87-MG, U251, A172, U373) and a patient-derived GBM primary culture (GL18-15).

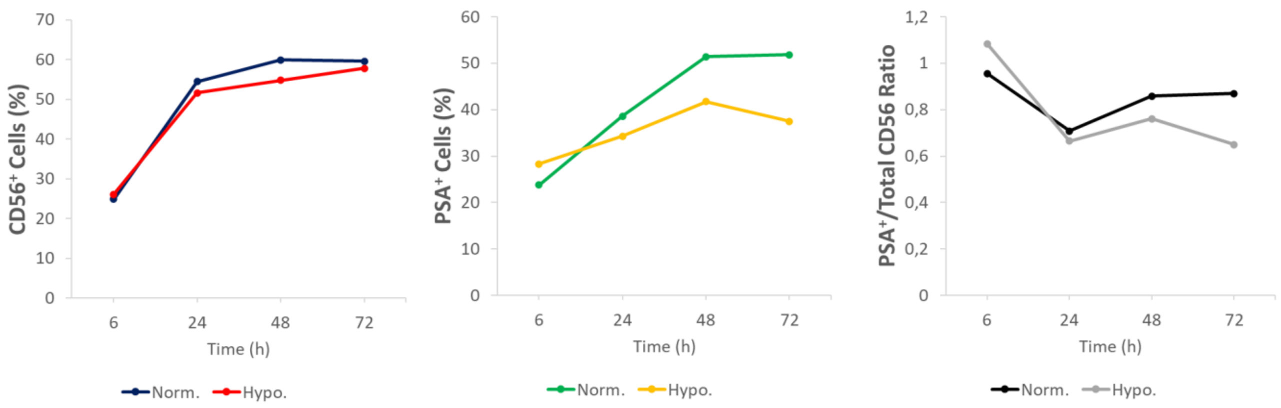

Figure S2 – Effects of the hypoxic microenvironment on the extracellular expression of NCAM and PSA in U87-MG cells. Cytofluorimetric analysis showing the time course (0-72h) of expression of NCAM (indicated as CD56, left panel) and PSA (middle panel) on the extracellular side of U87-MG cells grown either under normoxia or hypoxia. The panel on the right reports the ratio PSA/total NCAM calculated for U87-MG cells under low and normal oxygen concentrations.

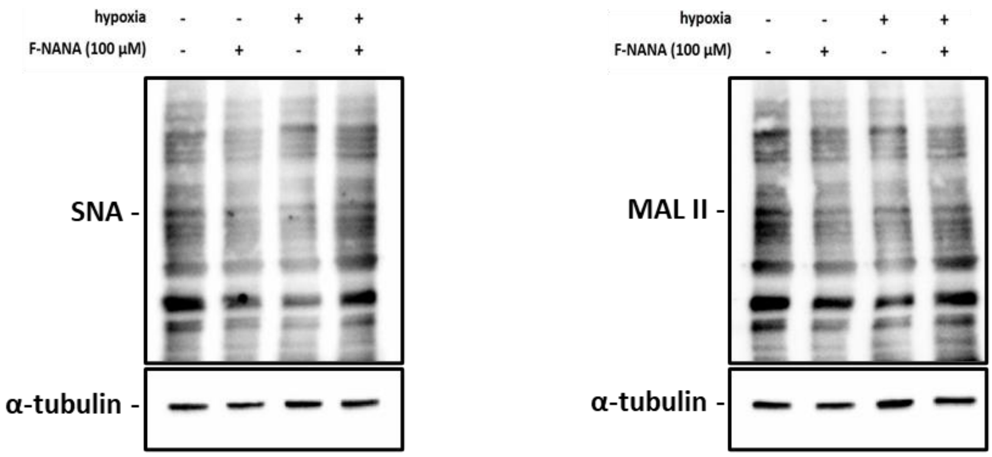

Figure S3 – Effects of F-NANA on the expression of the lectins SNA and MAL II in U87-MG cells under hypoxia.

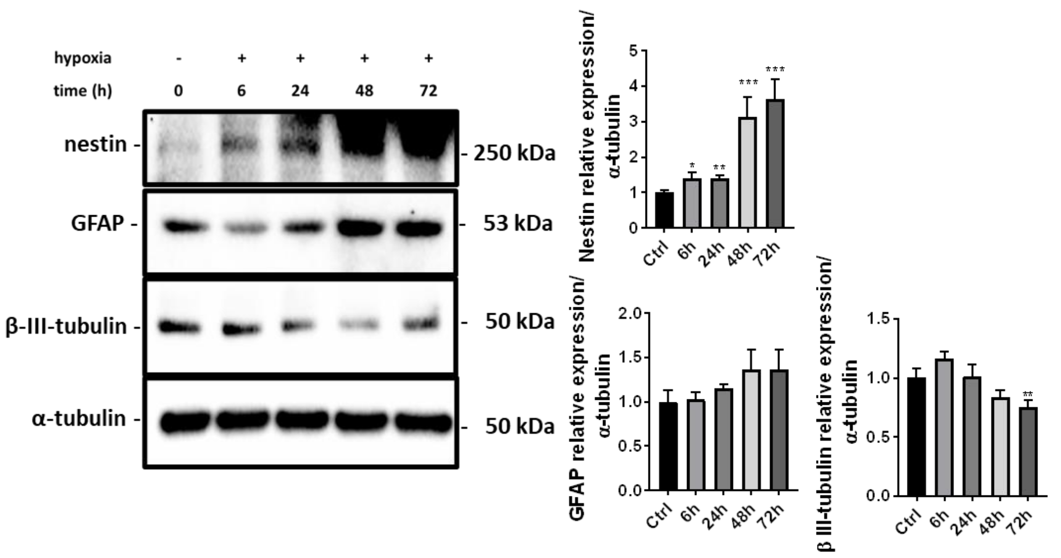

Figure S4 – Expression of stemness and differentiation markers in U87-MG cells under hypoxia. Western blot showing the expression of the stemness marker nestin and the differerentiation markers GFAP and βIII-tubulin in a time course of hypoxia (0-72h) in U87-MG cells.

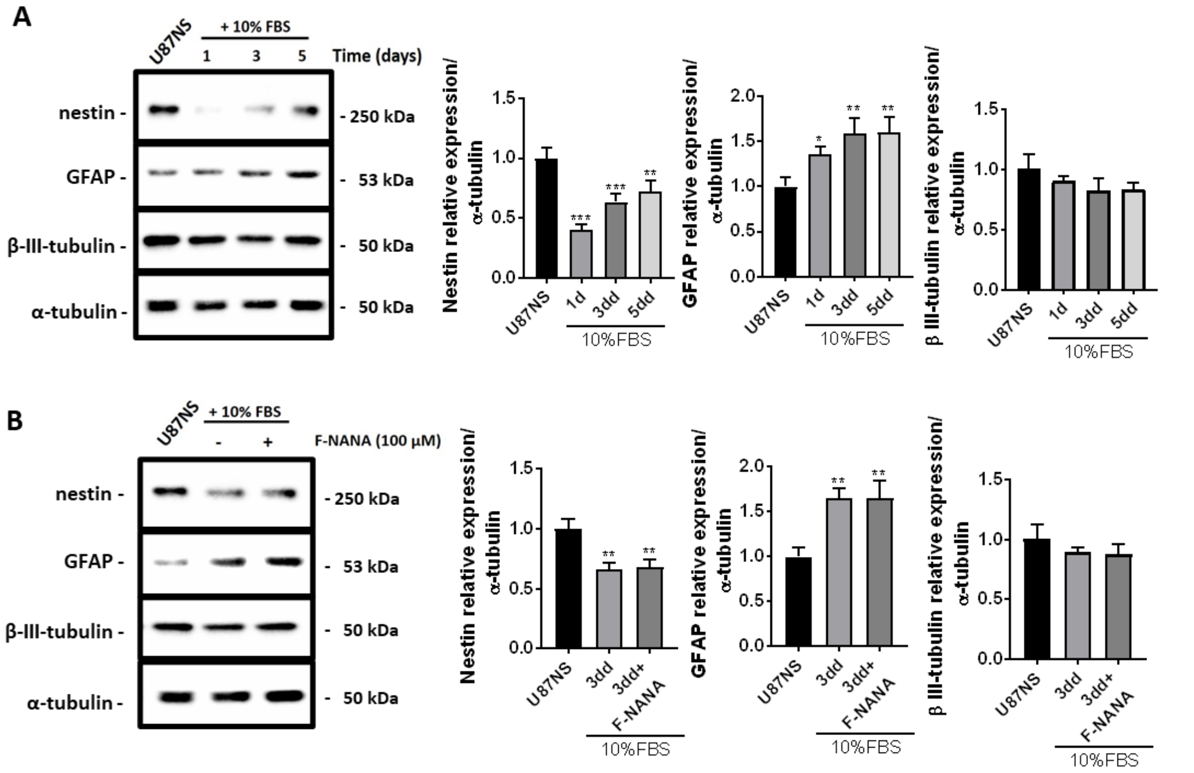

Figure S5 – Effects of F-NANA on the expression of Nestin, GFAP and βIII-tubulin in the differentiation of U87NS cells. A) Western blot showing the expression of the stemness marker nestin and the differentiation markers GFAP and βIII-tubulin in a time course (0-5 days) of differentiation of U87NS cells. **B)** Western blot showing the expression of nestin, GFAP and βIII-tubulin in U87NS differentiated or not for three days, in presence or not of 100μM of F
